# Integrating hydrology and biogeochemistry across frozen landscapes

**DOI:** 10.1038/s41467-019-13361-5

**Published:** 2019-11-26

**Authors:** J. E. Vonk, S. E. Tank, M. A. Walvoord

**Affiliations:** 1 Department of Earth Sciences, Vrije Universiteit Amsterdam, Amsterdam, The Netherlands; 2 Department of Biological Sciences, University of Alberta, Edmonton, AB Canada; 3 Earth System Processes Division, United States Geological Survey, Lakewood, USA

**Keywords:** Biogeochemistry, Hydrology

## Abstract

Research has traditionally focused on atmospheric release of carbon from thawing permafrost, yet overlooked waterborne release pathways likely contribute significantly, especially in a warming Arctic. To address this knowledge gap and better constrain the fate of carbon in the North, we recommend inter-disciplinary efforts bridging physical, chemical and computational research.

## Abstract (web summary)

As climate change thaws the Arctic’s foundations, new subterranean waterways form and threaten to wash away and decompose carbon once locked in permafrost. In this Comment, Vonk and co-authors outline a cross-disciplinary strategy--with hydrology at the forefront--to better understand the fate of Arctic carbon.

Change is unfolding beneath the surface of frozen landscapes that affects the coupled water and carbon cycles, and thus the fate of permafrost carbon, at local to pan-arctic scales. Degrading permafrost alters landforms and subsurface hydraulic properties, which allows water to infiltrate and circulate more freely and deeply^1,2^. With thaw, organic matter is released from the large reservoir of permafrost carbon^3^ and may be converted into greenhouse gases via decomposition, giving rise to the permafrost carbon feedback that intensifies warming. While this direct route to the atmosphere is most commonly studied, organic matter is also mobilized into waterways. The form (e.g. gaseous, dissolved, particulate), pathway (Fig. 1), and magnitude of constituent release via waterways varies widely among different landscapes and hydrologic conditions^4^. Decomposition of organic matter in aquatic systems may generate greenhouse gas release downstream of the source, whereas aquatic systems may also be a sink for carbon burial, attenuating the permafrost carbon feedback^5^. At the global scale, this so-called lateral flux of carbon rivals the combined magnitude of the terrestrial and oceanic sink for anthropogenic CO_2_^6,7^. The contribution of lateral permafrost carbon mobilization to the changing Arctic carbon cycle is increasingly recognized^4,6,8,9^ but remains poorly quantified.

**Fig. 1:**
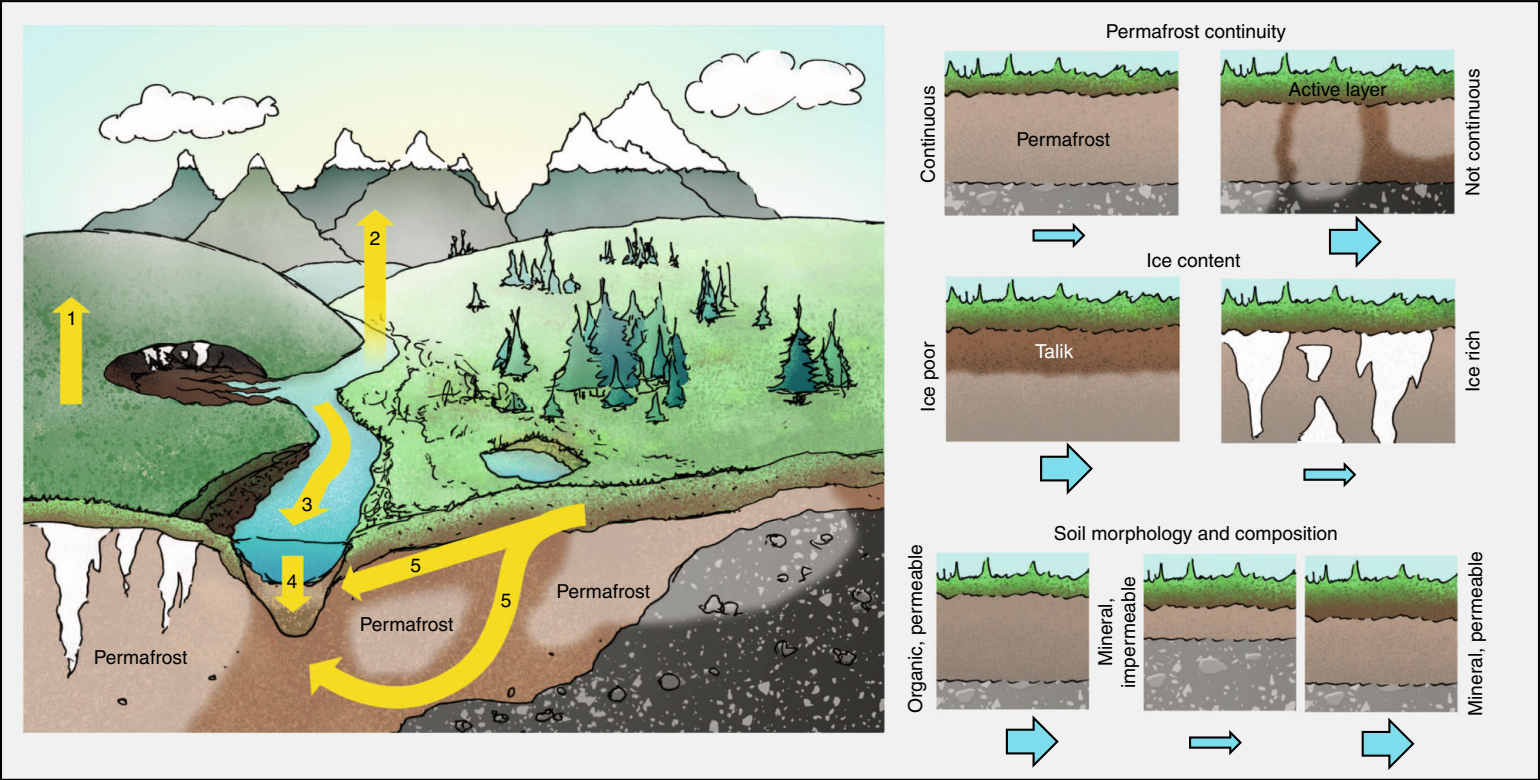
Landscape heterogeneity across the frozen north. The left panel illustrates a tundra plateau underlain with ice-rich permafrost that is vulnerable to thermokarst, versus a forested environment underlain with discontinuous ice-poor permafrost that is vulnerable to active layer deepening and enhanced subsurface connectivity with permafrost thaw. These contrasts in permafrost continuity and ice content, as well as the illustrated differences in soil composition and topography, generate a wide subsurface variability which controls the susceptibility, rate, and biogeochemical and hydrological consequences of thaw. Yellow arrows indicate five carbon pathways affected by thaw in an integrated terrestrial–aquatic continuum, with (1) terrestrial emission of greenhouse gases, (2) emission from waterways, (3) movement through inland and coastal waters, (4) sedimentary burial, and (5) leaching into and movement through porewater as illustrated. Current research tends towards isolated investigations of terrestrial (1), hydrologic (2), or aquatic (3–5) pathways, with a focus on the direct emission from terrestrial permafrost, i.e. only one of the five pathways illustrated here. The right schematics illustrate the contrasts in primary landscape factors with blue arrows indicating the relative subsurface hydrological permeability or potential for lateral aquatic C transport. Topography is a forcing factor that shapes all three of the landscape factors shown here.

Traditionally, lateral transport has been estimated from measurements taken in streams and rivers. In reality, however, the transport process begins within the soil. Soil pore water is a key variable in the generation and terrestrial emission of greenhouse gases from thawing permafrost (Fig. 1), but also serves as the genesis of water and constituent flow into inland or coastal waters. Studies provide mounting evidence of perennially thawed zones, or taliks, in permafrost across the northern hemisphere^8,10^. When laterally connective, taliks facilitate year-round subsurface mobilization and delivery of dissolved carbon to inland waters, altering the seasonality and magnitude of subsurface flow, and biogeochemical exports^8^. However, the lateral component of carbon transport, enhanced through talik development, remains neglected or handled as a residual in large-scale biogeochemical models^5^ that are spatially distributed 1-D (vertical) representations, treating aquatic and terrestrial domains as disconnected systems due to a combination of data, conceptual, and computational challenges. This disconnect will continue to impede advancement unless purposeful efforts are made to bridge the gap. Here we describe necessary steps for strengthening links between hydrology and biogeochemistry in data collection and modeling efforts to better identify and quantify impacts of thaw on landscapes and climate.

## A strategy for integrated hydrological–biogeochemical research

To maximize outcomes of our proposed inter-disciplinary approach, we propose research within the context of specific landscape factors that are critical determinants of thaw effects, targeting regions that are poised for the most immediate and greatest change. Intensive, site-based studies indicate that four factors—permafrost continuity, ice content, soil morphology, and topography (Fig. 1) determine the susceptibility, rate, and hydrologic and biogeochemical consequences of thaw^4^. The resolution and certainty of spatial and vertical data availability for these factors is however hugely variable. Future research focused on improving resolution and certainty of these factors will be critical for making robust predictions of thaw impacts across broad spatial scales.

Permafrost continuity influences soil permeability, flowpaths, and residence times of water. This factor also affects the quantity, composition, and age of mobilized carbon, together determining its ultimate fate. We have a poor understanding of permafrost extent, and its vertical distribution (i.e. depth to permafrost and total thickness) in regions of active thaw and in discontinuous terrains at depths > 1 m. This is particularly important because in these regions we expect the largest spatial heterogeneity in depth to permafrost, which determines hydrological connectivity upon thaw, as well as the potential exchange of water and solutes above and below permafrost.

Ice content also critically determines the type and rate of thaw, and accompanying shifts in permeability. Ice-poor soils with low thermal inertia may be more vulnerable to gradual thaw over larger spatial scales and release mostly dissolved constituents. Ice-rich soils, however, are prone to abrupt thaw and surface collapse predominantly releasing particulate constituents^11,12^. While localized, these abrupt processes may become regionally recognized through aggregation of localized occurrences as ubiquity increases^11^. Relatively poor information on ground ice content at the pan-arctic scale currently limits the accuracy of quantitative projections of region-specific thaw rates^2^ and thermokarst potential.

Soil composition and morphology are also important. This factor regulates permeability, which in turn affects water and solute fluxes, and transit times. Soil composition and age^13^ determine the constituent leaching yield^14^, the degree of mineral sorption of organic material, and molecular structure of the organic matter—factors that affect the quantity and fate of carbon that is liberated. Information on soil composition is, however, mostly confined to the 0–3 m depth range^3^, resulting in uncertainty in quantifying hydrological and biogeochemical consequences of evolving flowpaths and talik formation in degrading, deeper, permafrost.

Finally, topography (i.e. relief) is a forcing factor that regulates response to thaw, superimposed on intrinsic properties described above. Topography affects the long-term build-up of organic material and ground ice, while also influencing thermokarst development (landscape collapse when ice-rich permafrost thaws) and the potential for lateral transport of dissolved and particulate material. Greater relief generates greater hydraulic gradients which increases water fluxes, reduces transit time, and enhances the propensity for lateral carbon transport along the terrestrial–aquatic continuum. While a high-resolution Digital Elevation Model is recently available for land north of 60°N (www.pgc.umn.edu/data/arcticdem/), subsidence in thermokarst-susceptible regions may necessitate periodic updates.

## Next steps

We call for a calculated pivot in Arctic permafrost carbon research. The inter-disciplinary approach we recommend will move the field towards quantifying waterborne pathways of lateral carbon transport through integration of field-based data collection, and refined coupled hydrologic and biogeochemical transport models. Further, the continued development and field implementation of biogeochemical tracers can be used to infer changes in hydrology, with an eye to the critical, controlling nature of the factors described above. We suggest targeting regions near the continuous–discontinuous permafrost transition, and those experiencing substantial thermokarst. These high-priority regions are most susceptible to thaw-induced change in carbon liberation, via movement of water through activation of deep flowpaths, and the excavation of large volumes of previously frozen soils. In these key regions, systematic analysis of the factors outlined above will help focus computationally expensive modeling efforts, where lateral transport processes are most important and dynamic.

To keep pace with rapid advances in near-surface characterization and monitoring of permafrost landscapes through satellite remote sensing, we advocate complementary research toward subsurface characterization. Within the continuous–discontinuous transition zones we recommend to characterize subsurface high-resolution permafrost distribution, ice content, and soil composition (both chemical and physical), as these factors will determine how flowpaths will change, and the amount of permafrost carbon that will be transported as a result. For regions experiencing substantial thermokarst, our recommended focus is on characterization of ice content and soil composition at depth, in addition to topography. Though surface change detection provides useful information on landscape change that has already occurred, thaw often establishes itself at depth and therefore understanding subsurface properties provides a framework for predicting future thaw type, rate, and response. Complementary surface and subsurface approaches, including ground and airborne geophysical methods for multi-scale characterization^2^, will help capture the rapid but invisible change that is occurring underground at points of permafrost transition, and enable a better understanding of how the pronounced hydrologic–biogeochemical effects of thermokarst are likely to evolve.

A promising parallel direction to quantify waterborne release of permafrost carbon is to track permafrost thaw across multiple scales, from watersheds to large basins, through use of conservative biogeochemical tracers or proxies. Several tracers have proven valuable to visualize spatial or temporal patterns of thaw, for example molecular marker-based or carbon isotope-based source apportionment^15^. While each tracer has potential drawbacks, they show great promise for broader monitoring and advancing our understanding of the progression of thaw.

This call for an interdisciplinary way forward requires a specific focus on the primary landscape factors described above, ensuring that their effect on hydrology and biogeochemistry are considered where good data exist, and that efforts to improve data quality and coverage are focused towards the high-priority regions identified above. Given that these factors act as primary gate-keepers for the manner in which lateral flowpaths—and thus the connectivity of terrestrial and aquatic systems—are changing in our warming north, we urge their quantification and incorporation into field-based and modeling studies alike.

The pronounced effects of permafrost thaw on societal and Earth systems is without question. However, there is still much work to be done to quantify the carbon cycle effects of thaw. Here, we outline actionable priorities that will help determine how, and how much, carbon is liberated from landscapes across the variable north. This task—like the Arctic itself —is vast. However, implementing these recommendations will enable closing the gap imposed by lateral carbon transport on the changing northern carbon cycle. Such efforts are critical to understanding, and mitigating, the physical, ecological, and societal change being imposed on our planet’s north.
